# Transcriptional profiling of two contrasting genotypes uncovers molecular mechanisms underlying salt tolerance in alfalfa

**DOI:** 10.1038/s41598-021-84461-w

**Published:** 2021-03-04

**Authors:** Rakesh Kaundal, Naveen Duhan, Biswa R. Acharya, Manju V. Pudussery, Jorge F. S. Ferreira, Donald L. Suarez, Devinder Sandhu

**Affiliations:** 1 Department of Plants, Soils and Climate/Center for Integrated BioSystems, College of Agriculture and Applied Sciences, Utah State University, Logan, UT 84322 USA; 2 US Salinity Lab (USDA-ARS), 450 W Big Springs Road, Riverside, CA 92507 USA; 3 College of Natural and Agricultural Sciences, University of California Riverside, Riverside, CA 92507 USA

**Keywords:** Plant genetics, Gene expression

## Abstract

Alfalfa is an important forage crop that is moderately tolerant to salinity; however, little is known about its salt-tolerance mechanisms. We studied root and leaf transcriptomes of a salt-tolerant (G03) and a salt-sensitive (G09) genotype, irrigated with waters of low and high salinities. RNA sequencing led to 1.73 billion high-quality reads that were assembled into 418,480 unigenes; 35% of which were assigned to 57 Gene Ontology annotations. The unigenes were assigned to pathway databases for understanding high-level functions. The comparison of two genotypes suggested that the low salt tolerance index for transpiration rate and stomatal conductance of G03 compared to G09 may be due to its reduced salt uptake under salinity. The differences in shoot biomass between the salt-tolerant and salt-sensitive lines were explained by their differential expressions of genes regulating shoot number. Differentially expressed genes involved in hormone-, calcium-, and redox-signaling, showed treatment- and genotype-specific differences and led to the identification of various candidate genes involved in salinity stress, which can be investigated further to improve salinity tolerance in alfalfa. Validation of RNA-seq results using qRT-PCR displayed a high level of consistency between the two experiments. This study provides valuable insight into the molecular mechanisms regulating salt tolerance in alfalfa.

## Introduction

Salinity is one of the major stress factors that plants encounter in nature. More than 800 million hectares of irrigated land is affected by soil salinity, which is expected to increase further due to global climate change and current irrigation practices^1^. Salt stress is a significant factor limiting crop productivity worldwide. Some basic understanding has been established on various traits and processes involved in salinity tolerance (such as osmotic stress, ion exclusion, and tissue tolerance) in various crop plants^2,3^. However, an in-depth understanding of various component traits involved in salinity tolerance is warranted to develop salt-tolerant varieties that can meet increasing global food and feed demands.

Negative effects of salinity on plant growth result from osmotic stress, accumulation of Na^+^ and/or Cl^-^ in plant tissues to cytotoxic levels, mineral imbalance, and oxidative stress resulting from the excessive accumulation of reactive oxygen species (ROS) triggered by salinity^4,5^. During evolution, plants have developed mechanisms to adapt to salinity, which are thought to be of mainly three types^2^: calcium-mediated tolerance to osmotic shock, transporter proteins-mediated exclusion of salt ions harmful to cellular metabolic processes, and activity of vacuolar Na^+^/H^+^ antiports^6^. However, different plant species respond differently to salinity stress. Thus, there is a need for a more comprehensive molecular study of alfalfa response to salinity to help in identifying and generating new salt-tolerant lines that can be used in breeding programs.

Alfalfa is the most widely cultivated perennial forage legume in the world due to its high protein content and palatability to livestock. It is cultivated on more than 23 million acres in the United States alone^7^. Compared to other legume crops, alfalfa is moderately tolerant to salt stress^2,5^; nonetheless, salinity stress is an important factor that is responsible for the lower yield of alfalfa. Due to the importance of alfalfa for the dairy industry and the availability of tremendous genetic variability in the germplasm, increasing salt tolerance in alfalfa has a great economic potential^8^. Recent isolation and characterization of genes triggered by abiotic stress have provided some insight into salinity tolerance in alfalfa^9^. Several approaches like germplasm selection, in vitro selection, marker-assisted selection (MAS), and transgene expression helped to recognize the role of different genes during salinity stress in alfalfa^10–12^. A better understanding of the mechanisms of salt-tolerance in alfalfa can be generated by employing cutting-edge technologies such as transcriptomics^13,14^ and proteomics^15^.

In alfalfa, root transcriptome in response to salinity stress during germination^16^ and leaf transcriptome in response to salinity stress^17^ have been studied individually. However, a comprehensive study of root and leaf transcriptome analyses in response to long-term salinity stress in adult plants is still missing in alfalfa. Although a high sequence similarity and conserved genome structure are shared by *M. truncatula* and *M. sativa,* information obtained from *M. truncatula* reference genome is not always useful in *M. sativa*^16^. Because closely-related species are known to vary in gene expression patterns, the study of alfalfa-specific expression will benefit from the de novo sequencing of the alfalfa genome, which will help to identify key genes involved in salt tolerance in alfalfa.

In this study, we explored the differential gene expression between two contrasting alfalfa genotypes [SISA14-1 (G03)—a salt-tolerant genotype and SISA10 (G09)—a salt-sensitive genotype] at the maturity stage by transcriptional profiling of root and leaf tissues of plants kept under long-term irrigation with either high- or low-salinity water (control) treatments. The main objective of this study was to identify candidate genes involved in different component traits of the salt tolerance mechanisms in alfalfa. The selected candidate genes can be explored further for their specific role during salinity tolerance in alfalfa and utilized in breeding programs to develop new salt-tolerant varieties.

## Results

### De novo transcript assembly and functional annotation

In order to gain a general insight into gene expression profiles of two alfalfa genotypes (G03, salt-tolerant and G09, salt-sensitive) for roots (R) and leaves (L) under salt stress, 24 cDNA libraries were designed for RNA sequencing, representing control treatments (C) as C03L, C03R, C09L, and C09R and salt treatments (T) as T03L, T03R, T09L, and T09R in three replicates (Supplementary Table S1). Supplementary Fig. S1 shows the overall workflow of our transcriptome analysis. A total of 1,739,103,320 high-quality reads consisting of 260.85 Gbases were obtained after discarding low-quality sequences, ambiguous nucleotides, and adapter sequences from 1,807,829,788 total raw reads. For each library, reads consisting of at least 6.0 Gbases were obtained (Supplementary Table S2). In total, 421,608 transcripts (≥ 200 bp) were obtained with Trinity de novo assembler, and with transcript sizes ranging from 200 to 10,811 with a mean size of 750 bp. Out of 421,608 transcripts, 23% (97,926) were longer than 1,000 bp (Supplementary Fig. S2). In total, 418,480 unigenes (≥ 200 bp) with a mean length of 753 bp were clustered using Corset (https://github.com/Oshlack/Corset/wiki). For the functional annotation of all unigenes, searches against seven public databases showed a significant similarity of unigenes with the sequences in these databases ranging from 65,987 (15.77% in KOG (euKaryotic Orthologous protein Groups) to 327,517 (78.26% in NT (NCBI non-redundant nucleotide sequences) (Supplementary Fig. S3). Of all unigenes, 348,019 (83.16%) were annotated in at least one database and 32,762 (7.83%) in all seven databases (Supplementary Fig. S3).

In total, 147,569 (35%) unigenes were assigned to 57 gene ontology (GO) level-2 annotations and classified into three main groups, biological process (BP), cellular component (CC), and molecular function (MF) (Supplementary Fig. S4, Supplementary Table S3). Further, all the unigenes were assigned to the Kyoto Encyclopedia of Genes and Genomes (KEGG) and KOG pathway databases. In the KEGG database, 56,888 genes were annotated in 130 KEGG pathways (Supplementary Fig. S5), whereas in KOG, 65,987 unigenes were assigned to 26 functional classes (Supplementary Fig. S6).

### Gene expression

**Figure 1:**
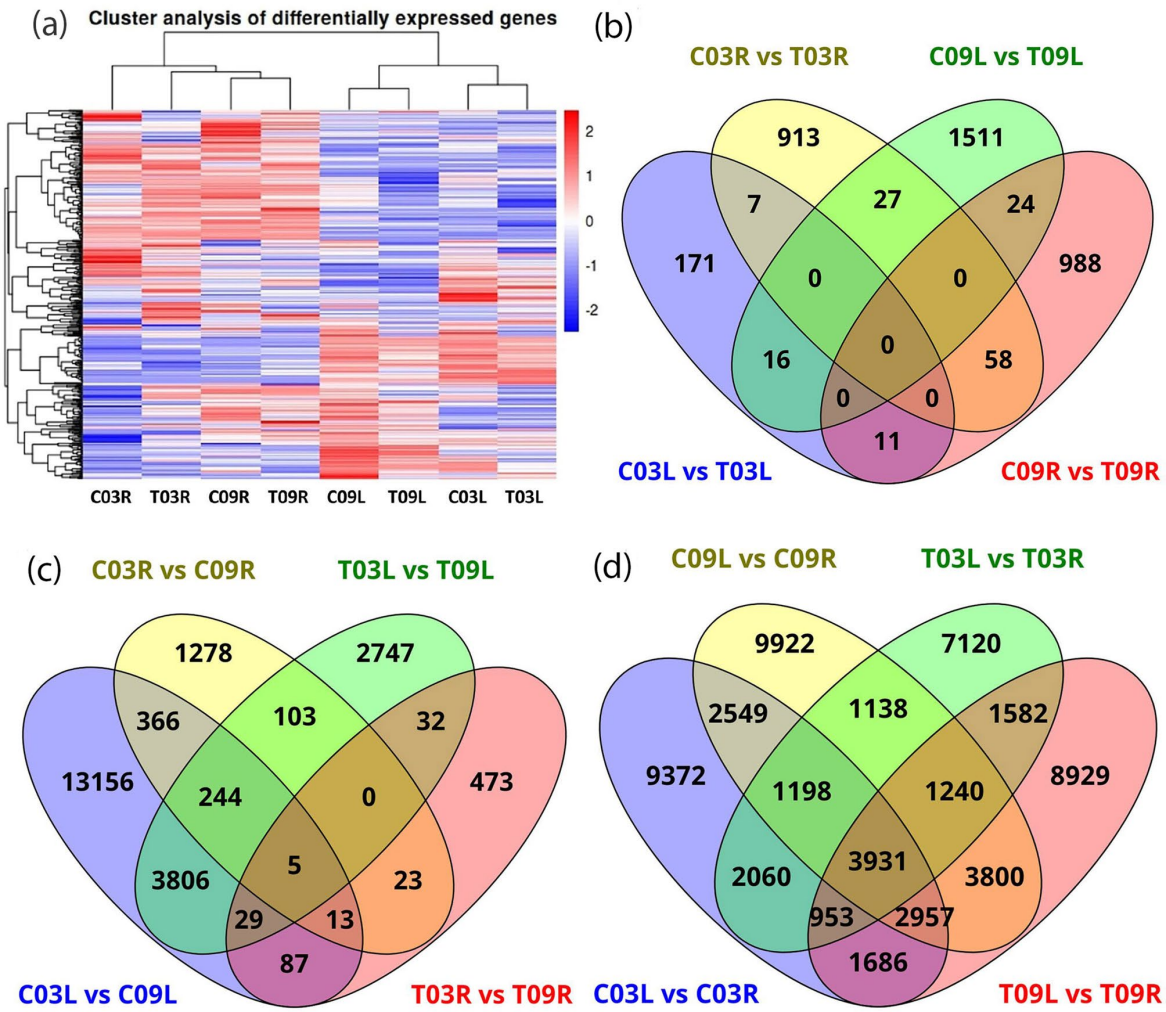
**(a)** A heatmap showing expression patterns of differentially expressed genes across eight samples, **(b)** Venn diagram representing the number of differentially expressed genes across four control vs. salt-treatment comparisons and the overlap between each set of genes, **(c)** Venn diagram representing the number of differentially expressed genes across four salt-tolerant vs. salt-sensitive comparisons and the overlap between each set of genes, **(d)** Venn diagram representing the number of differentially expressed genes across four leaf vs. root comparisons and the overlap between each set of genes.

Gene expression levels of different samples were calculated by mapping reads of each replicate against the assembled transcriptome; a total of 72.65% reads mapped onto the assembly (Supplementary Table S2). Cluster analysis of the mapped reads showed the presence of two major groups. The first group consisted of the unigenes present in both control (C) and treatment (T) groups of root transcriptome (R) while the second group consisted of unigenes in control and treatment groups of leaf (L) transcriptome (Fig. 1a). Genes from C09R and T09R cluster formed a sub-group, while C03R clustered with T03R separately within the first group. In the second group, two sub-groups formed: one with C03L and T03L clustered in one sub-group and the other with C09L and T09L clustered together (Fig. 1a).

### Differentially expressed genes (DEGs) in response to salt stress

Results are organized in three different comparisons: (a) control vs. salt treatment, (b) salt-tolerant vs. salt-sensitive genotype, and (c) leaf vs. root. Out of 418,480 unigenes, 68,847 (16.45%) were differentially expressed (at fold change ≥ 2 and FDR ≤ 0.05) in at least one of the comparisons. The smallest number of DEGs were detected in control vs. salt treatment comparisons (Table 1). In the control vs. salt-treatment, there were 205 DEGs in C03L vs. T03L (149 upregulated and 56 down-regulated); 1,578 DEGs in C09L vs. T09L (893 upregulated and 685 down-regulated); 1,005 DEGs in C03R vs. T03R (522 upregulated and 483 down-regulated); and 1,081 DEGs in C09R vs. T09R (557 upregulated and 524 down-regulated) (Table 1, Fig. 1b). In the salt-tolerant vs. salt-sensitive comparison, 17,706 genes were differentially expressed in C03L vs. C09L (9,080 upregulated and 8,626 downregulated); 2,032 in C03R vs. C09R (1008 upregulated and 1,024 downregulated); 6,966 in T03L vs. T09L (2,335 upregulated and 4,631 downregulated); and 662 in T03R vs. T09R (311 upregulated and 351 downregulated) (Table 1, Fig. 1c). In leaf vs. root comparisons, a total of 24,706 genes were found to be differentially expressed in C03L vs. C03R comparison (13,964 upregulated and 10,742 down-regulated); 26,735 DEGs in C09L vs. C09R (13,850 upregulated and 12,885 down-regulated); 19,222 DEGs in T03L vs. T03R (7,769 upregulated and 11,453 down-regulated); 25,078 in T09L vs. T09R (13,994 upregulated and 11,084 down-regulated) (Table 1, Fig. 1d).

**Table 1:** Differentially expressed genes (DEGs) identified in different comparisons.

Comparison	Groups	DEGs	Upregulated	Downregulated
Control vs. salt	C03L vs. T03L	205	149	56
	C09L vs. T09L	1578	893	685
	C03R vs. T03R	1005	522	483
	C09R vs. T09R	1081	557	524
Salt-tolerant vs. salt-sensitive	C03L vs. C09L	17,706	9080	8626
	T03L vs. T09L	6966	2335	4631
	C03R vs. C09R	2032	1008	1024
	T03R vs. T09R	662	311	351
Leaf vs. root	C03L vs. C03R	24,706	13,964	10,742
	C09L vs. C09R	26,735	13,850	12,885
	T03L vs. T03R	19,222	7769	11,453
	T09L vs. T09R	25,078	13,994	11,084

*C* control, *T* treatment, *03* salt-tolerant genotype, *09* salt sensitive genotype; *L* leaf, *R* root.

In four control vs. salt treatment comparisons, DEGs unique to a particular comparison were 171, 913, 1,511, and 988 for C03L vs. T03L, C03R vs. T03R, C09L vs. T09L, and C09R vs. T09R, respectively. Interestingly, none of the DEGs were common among the four comparisons (Fig. 1b and Supplementary Table S4).

In four salt-tolerant vs. salt-sensitive comparisons, DEGs unique to a particular comparison were 13,156, 1,278, 2,747, 473 for C03L vs. C09L, C03R vs. C09R, T03L vs. T09L and T03R vs. T09R, respectively (Fig. 1c). Only 5 DEGs were common among the four comparisons (Clusters—49084.119239, 49084.126033, 49084.22008, 49084.127631, and 49084.156760). These clusters maintained similar fold change values in all four comparisons (Supplementary Table S4). Currently, the biological functions of these clusters during salinity stress are unknown.

In four comparisons of leaf vs. root, DEGs unique to pertinent comparisons were 9372, 9922, 7120 and 8929 for C03L vs. C03R, C09L vs. C09R, T03L vs. T03R, and T09L vs. T09R, respectively (Fig. 1d and Supplementary Table S4), while a total of 3931 DEGs were common among all the four comparisons.

### Gene ontology (GO) enrichment analysis of differentially expressed genes (DEGs)

**Figure 2:**
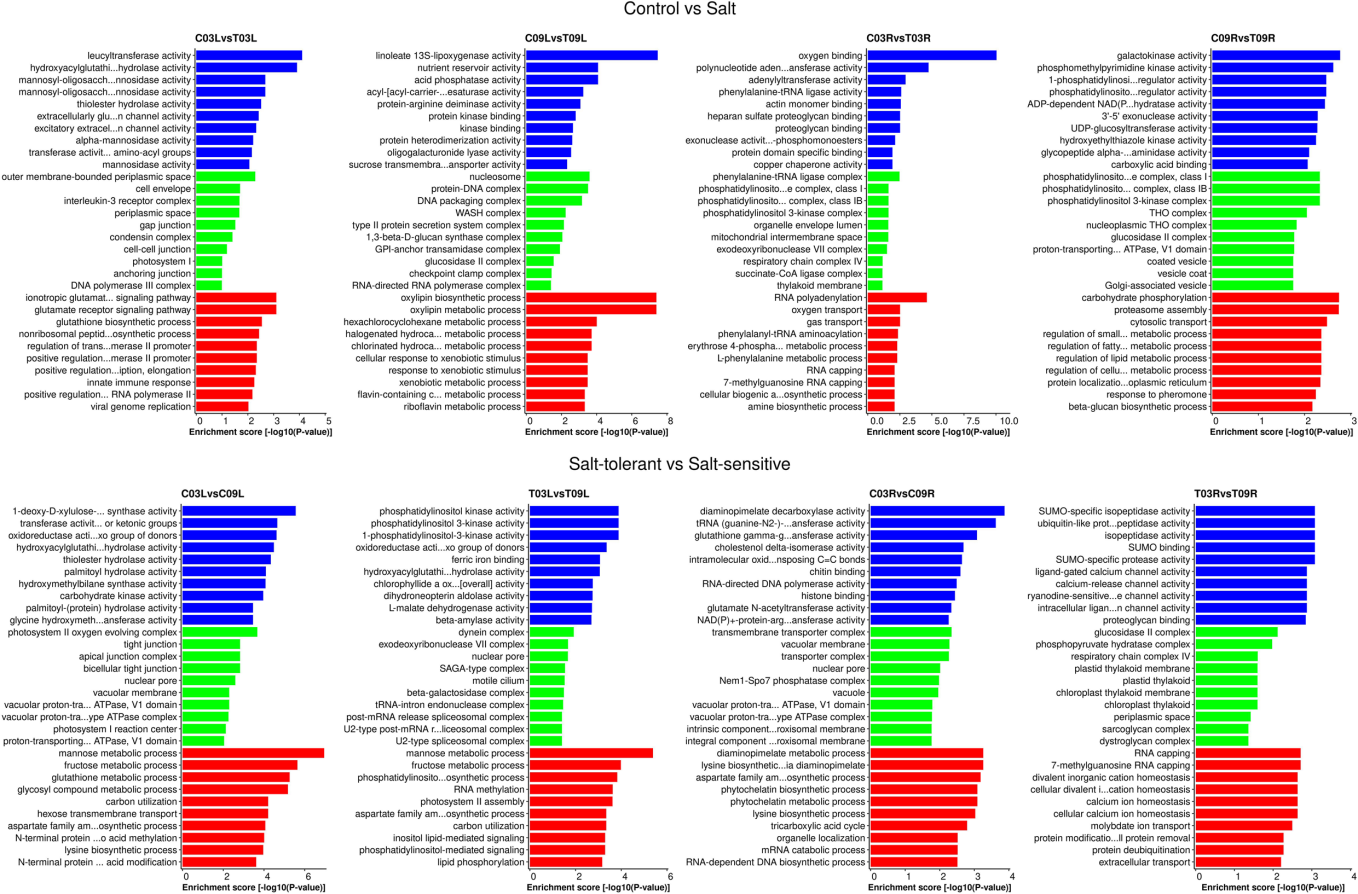
Gene ontology (GO) enrichment analyses of C03L vs. T03L, C09L vs. T09L, C03R vs. T03R, C09R vs. T09R, C03L vs. C09L, T03L vs. T09L, C03R vs. C09R, and T03R vs. T09R. For each comparison, blue color represents Molecular Function (MF), green represents Cellular Component (CC) and red color represents Biological Process (BP). Only the top 10 GO terms based on enrichment score (− log_10_[P-value]) for each GO category are shown in the figure.

Functional enrichment analysis was performed to study DEGs further. Three major categories of GO enrichment analysis (molecular function, MF; cellular component, CC; and biological process, BP) were considered. In the control versus salt treatment comparisons, GO terms significantly enriched in C03L vs. T03L include leucyltransferase activity (GO:0008914), outer membrane-bound periplasmic space (GO:0030288), and ionotropic glutamate receptor signaling pathway (GO:0035235), whereas GO terms such as oxygen binding (GO:0019825), phenylalanine-tRNA ligase complex (GO:0009328), and RNA polyadenylation (GO:0043631), were found to be enriched and over-represented in C03R vs. T03R (Fig. 2 and Supplementary Table S5); while GO terms enriched and over-represented in C09L vs. T09L were linoleate 13S-lipoxygenase activity (GO:0016165), nucleosome (GO:0000786), and oxylipin metabolic process (GO: 0031407), GO terms like galactokinase activity (GO:0004335), phosphatidylinositol 3-kinase complex, class IB (GO:0005944), and carbohydrate phosphorylation (GO:0046835) were found to be significantly enriched in C09R vs. T09R (Fig. 2 and Supplementary Table S5). In the salt-tolerant versus salt-sensitive comparisons, in C03L vs. C09L, the enriched and over-represented GO terms include 1-deoxy-d-xylulose-5-phosphate synthase activity (GO:0008661), photosystem II oxygen-evolving complex (GO:0009654), and mannose metabolic process (GO:0006013), whereas GO terms consisting of terms such as diaminopimelate decarboxylase activity (GO:0,008,836), transmembrane transporter complex (GO:1902495), and diaminopimelate metabolic process (GO:0046451) were found to be enriched in C03R vs. C09R (Fig. 2 and Supplementary Table S5). In T03L vs. T09L comparison, terms such as phosphatidylinositol 3-kinase activity (GO:0035004), dynein complex (GO:0030286), and mannose metabolic process (GO:0006013) were enriched and over-represented, whereas GO terms including SUMO-specific isopeptidase activity (GO:0070140), glucosidase II complex (GO:0017177), and RNA capping (GO:0036260) were enriched and over-represented in T03R vs. T09R (Fig. 2 and Supplementary Table S5).

### KEGG pathway analysis

**Figure 3:**
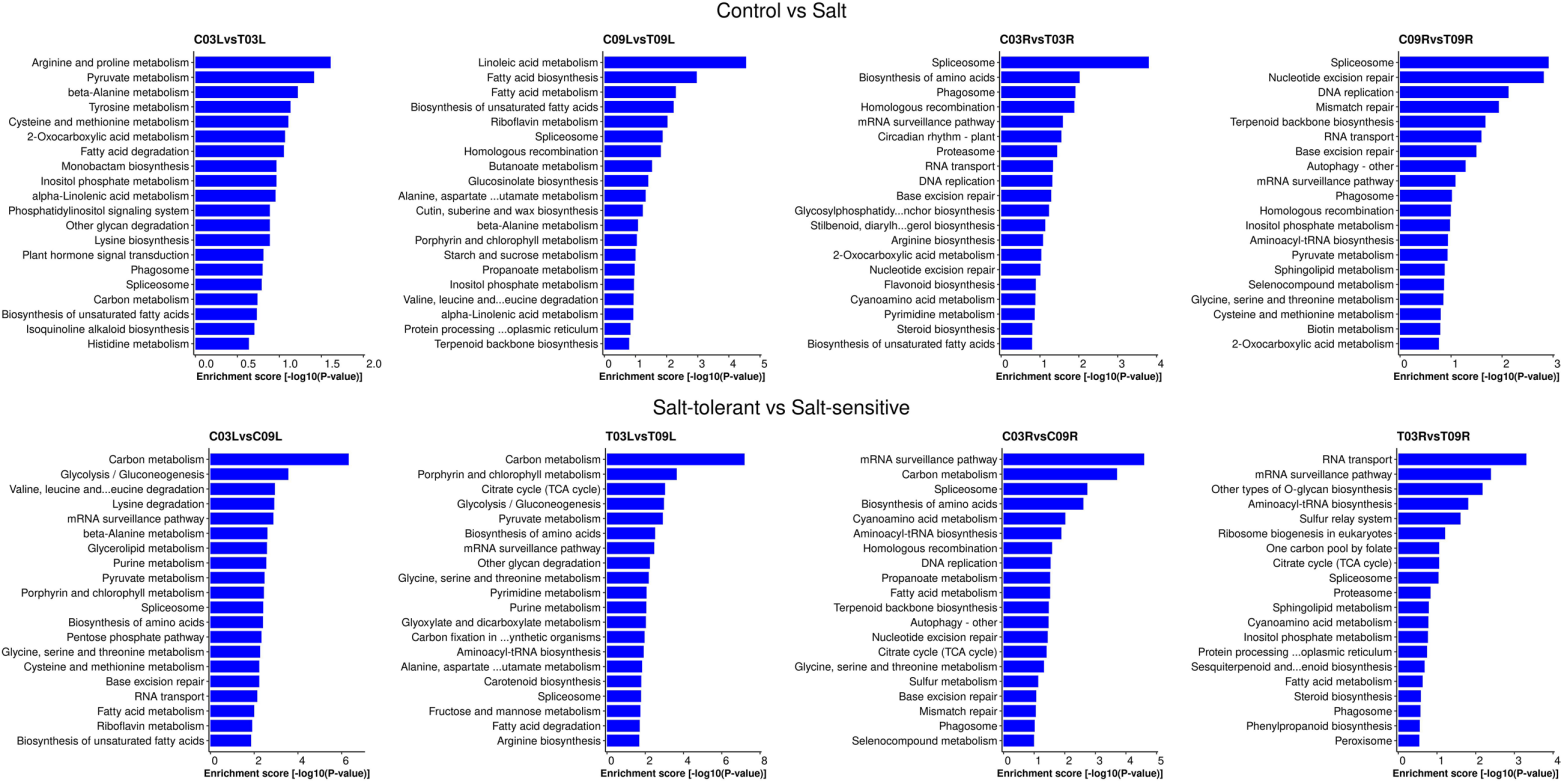
Kyoto Encyclopedia of Genes and Genomes (KEGG) pathway enrichment analyses of C03L vs. T03L, C09L vs. T09L, C03R vs. T03R, C09R vs. T09R, C03L vs. C09L, T03L vs. T09L, C03R vs. C09R, and T03R vs. T09R. For each pairwise comparison, only the top 20 enriched pathways based on enrichment score (− log_10_[P-value]) are shown in the figure.

To characterize the complex behavior of the alfalfa transcriptome, all the DEGs were subjected to a KEGG pathway enrichment analysis. In the control vs. salt treatment comparisons, pathways like arginine and proline metabolism (mtr00330) and pyruvate metabolism (mtr00620) were enriched in C03L vs. T03L, whereas pathways such as spliceosome (mtr03040) and biosynthesis of amino acids (mtr01230) were enriched in C03R vs. T03R; while C09L vs. T09L was enriched with pathways like linoleic acid metabolism (mtr00591) and fatty acid biosynthesis (mtr00061), for the C09R vs. T09R comparison, pathways such as spliceosome (mtr03040) and nucleotide excision repair (mtr03420) were significantly enriched (Fig. 3 and Supplementary Table S6). In the salt-tolerant vs. salt-sensitive comparisons, the carbon metabolism pathway (mtr01200) was enriched in both C03L vs. C09L and T03L vs. T09L comparisons. On the other hand, the mRNA surveillance pathway (mtr03015) was enriched in both C03R vs. C09R and T03R vs. T09R comparisons (Fig. 3 and Supplementary Table S6). The complete list of enriched KEGG pathways can be found in the Supplementary Table S6 for each of the pairwise comparisons.

### Differentially expressed genes (DEGs) involved in stress-related pathways

In order to understand the key DEGs regulating salt stress, all DEGs were analyzed with MapMan. DEGs linked to some major stress-related pathways such as hormone biosynthesis and signal transduction, calcium signaling, and redox signaling were identified within the regulation function terms.

#### Hormone biosynthesis and signal transduction

**Figure 4:**
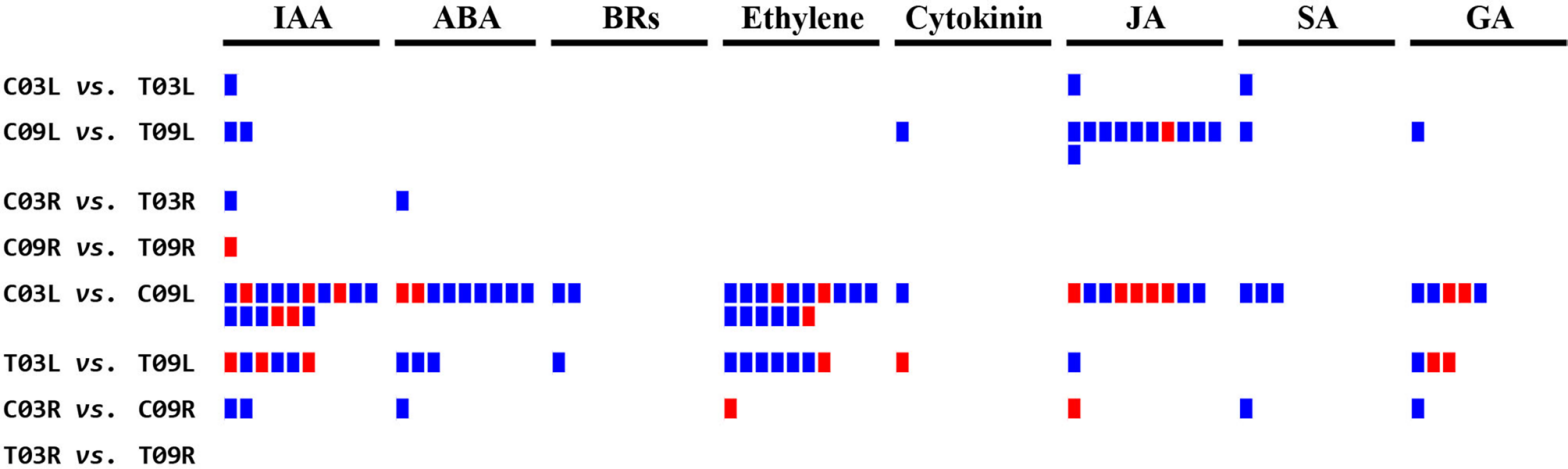
MapMan analysis of pairwise comparisons for genes associated with hormone biosynthesis and signal transduction. The blue color represents upregulation and the red represents downregulation. *IAA* indole acetic acid, *ABA* abscisic acid, *BRs* brassinosteroids, *JA* jasmonic acid, *SA* salicylic acid, *GA* gibberellins.

In the control vs. salt comparisons, 3 DEGs were involved in hormonal biosynthesis and signal transduction in C03L vs. T03L. Of these, 1 gene for indole-3-acetic acid (IAA), 1 gene for jasmonic acid (JA), and 1 gene for salicylic acid (SA) synthesis were found upregulated in C03L compared to T03L (Fig. 4 and Supplementary Table S7). For the C09L vs. T09L comparison, 16 genes involved in hormonal biosynthesis were upregulated in C09L compared to T09L, which included 2 genes for IAA, 1 gene for cytokinin, 10 genes for JA, 1 gene for SA and 1 gene for gibberellins (GA), whereas one gene for JA was downregulated (Fig. 4 and Supplementary Table S7). One DEG involved in IAA and one in abscisic acid (ABA) biosynthesis were upregulated in C03R compared to T03R (Fig. 4 and Supplementary Table S7). Only one DEG involved in IAA biosynthesis was downregulated in C09R compared to T09R (Fig. 4 and Supplementary Table S7).

In the salt-tolerant vs. salt-sensitive comparisons, of the 60 DEGs involved in hormone biosynthesis and signal transduction in C03L vs. C09L, 16 genes (11 upregulated and 5 downregulated) were for IAA, 9 genes (7 upregulated and 2 downregulated) for ABA, 2 genes (upregulated) for Brassinosteroids (BR), 16 genes (13 upregulated and 3 downregulated) for ethylene, 1 gene (upregulated) for cytokinin, 9 genes (4 upregulated and 5 downregulated ) for JA, 3 genes (upregulated) for SA and 5 genes (3 upregulated and 2 downregulated) for GA (Fig. 4 and Supplementary Table S7). Out of the 22 DEGs involved in the biosynthesis of hormones and signal transduction in T03L vs. T09L, 6 genes (3 upregulated and 3 downregulated) were for IAA, 3 genes (upregulated) for ABA, 1 gene (upregulated) for BR, 7 genes (6 upregulated and 1 downregulated) for ethylene, 1 gene (downregulated) for cytokinin, 1 gene (upregulated) for JA and 3 genes (1 upregulated and 2 downregulated) for GA (Fig. 4 and Supplementary Table S7). Seven DEGs for the C03R vs. C09R comparison were involved in hormone metabolism; 2 genes (upregulated) were for IAA, 1 gene (upregulated) for ABA, 1 gene (downregulated) for ethylene, 1 gene (downregulated) for JA, 1 gene (upregulated) for SA and 1 gene (upregulated) for GA (Fig. 4 and Supplementary Table S7). While in T03R vs. T09R, no DEGs were involved in the biosynthesis of hormones.

#### Calcium signaling

**Figure 5:**
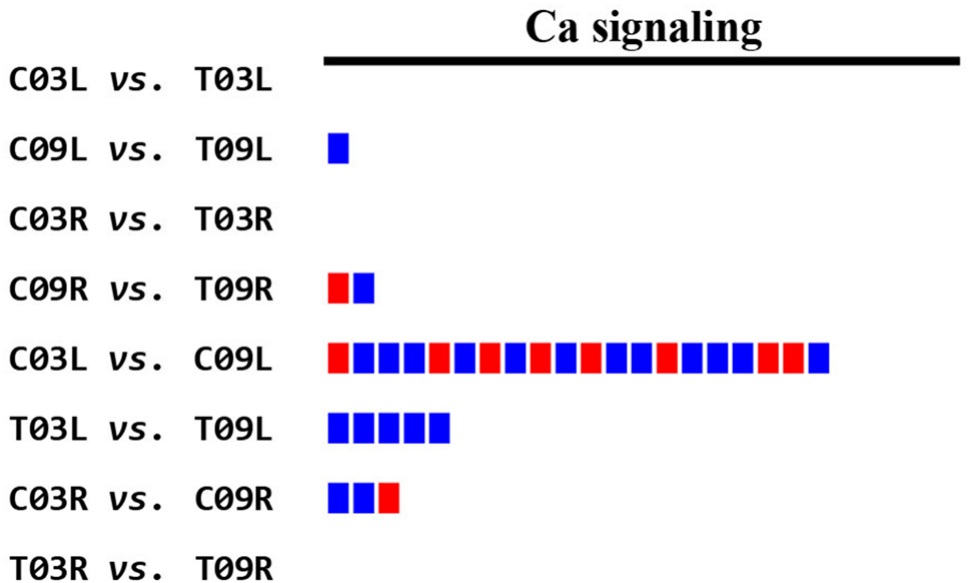
MapMan analysis of pairwise comparisons for calcium signaling pathway. The blue color represents upregulation and the red represents downregulation.

For the control vs. salt comparisons, 1 DEG involved in calcium signaling was upregulated in C09L as compared to T09L. In C09R vs. T09R, 2 DEGs (1 upregulated and 1 downregulated) were involved in calcium signaling (Fig. 5 and Supplementary Table S8). No DEGs involved in calcium signaling were found in the C03L vs. T03L and C03R vs. T03R comparisons (Fig. 5). For the salt-tolerant vs. salt-sensitive comparisons, in C03L vs. C09L, 21 DEGs were involved in calcium signaling, of which 13 were upregulated, and 8 were downregulated (Fig. 5 and Supplementary Table S8). Five DEGs involved in calcium signaling were upregulated in T03L compared to T09L (Fig. 5 and Supplementary Table S8). For the C03R vs. C09R comparison, 2 genes involved in calcium signaling were upregulated and one was downregulated (Fig. 5 and Supplementary Table S8). There were no DEGs involved in calcium signaling in T03R vs. T09R (Fig. 5).

#### Redox signaling pathway

**Figure 6:**
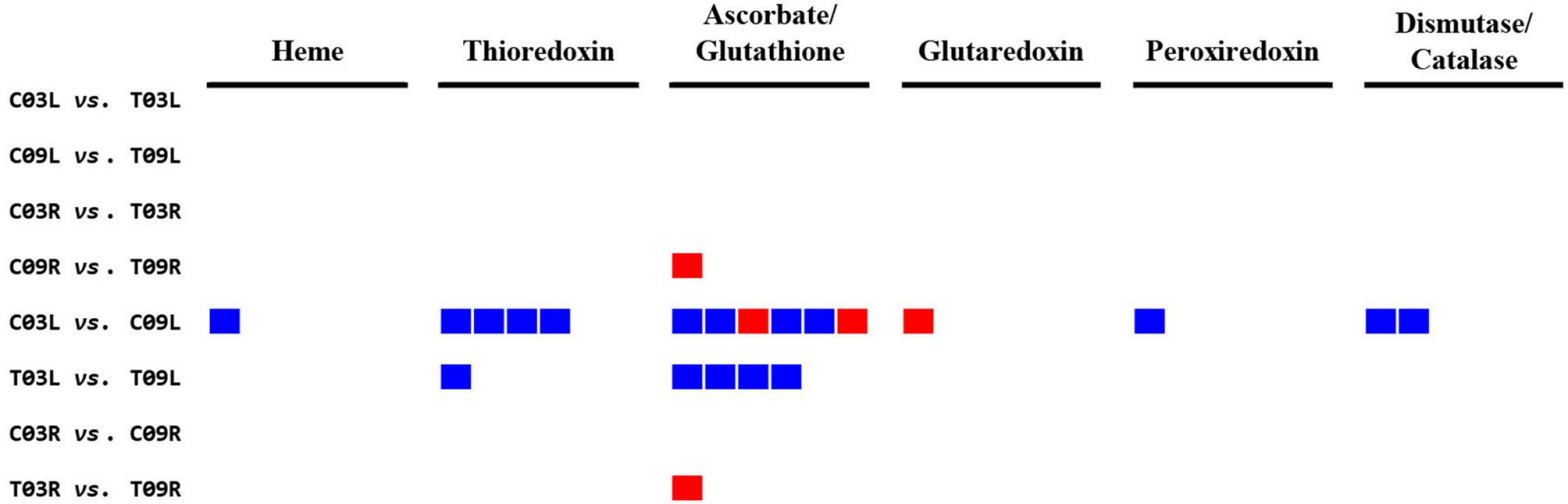
MapMan analysis of pairwise comparisons for the Redox signaling pathway. The blue color represents upregulation and the red represents downregulation.

In the control vs. salt treatment comparisons, there were no DEGs found regulating redox signaling pathway in C03L vs. T03L, C09L vs. T09L, and C03R vs. T03R, while 1 DEG encoding ascorbate/glutathione was downregulated in C09R vs. T09R for redox signaling (Fig. 6 and Supplementary Table S9). In the salt-tolerant vs. salt-sensitive comparisons, 15 DEGs regulating redox pathways were found in C03L vs. C09L; of these, 1 gene (upregulated) was for heme, 4 genes (upregulated) for thioredoxin, 6 genes (4 upregulated and 2 downregulated) for ascorbate/glutathione, 1 gene (downregulated) for glutaredoxin, 1 gene (upregulated) for peroxiredoxin and 2 genes (upregulated) were for dismutase/catalase (Fig. 6 and Supplementary Table S9). Five DEGs regulating redox signaling were found in T03L vs. T09L, out of which 1 gene (upregulated) was for thioredoxin and 4 genes (upregulated) for ascorbate/glutathione. For C03R vs. C09R, no DEGs represented the redox signaling pathway while 1 DEG was downregulated for ascorbic acid in T03R vs. T09R (Fig. 6 and Supplementary Table S9).

#### Verification of the DEGs using quantitative reverse transcription-polymerase chain reaction (qRT-PCR)

**Figure 7:**
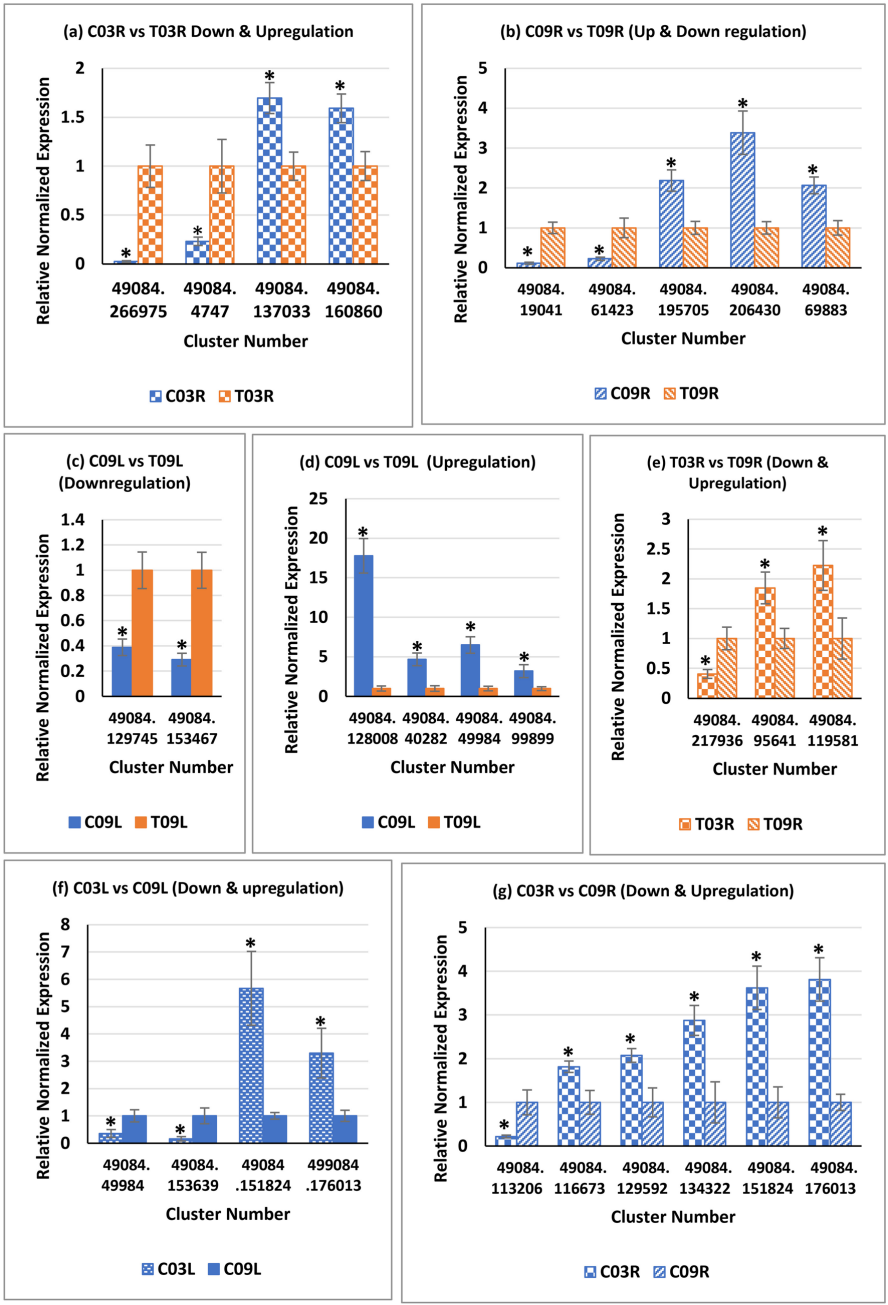
Validation of expression differences observed in RNA-seq data through quantitative Reverse Transcription-PCR. Expression status of alfalfa genes in the root (R), leaf (L) in two alfalfa genotypes [G03 (SISA 14-1) and G09 (SISA 10)] under control (C) and saline (T) treatments. Error bars represent standard error. An asterisk indicated a significant difference (P ≤ 0.05).

To verify the RNA-seq results, a total of 25 differentially expressed alfalfa clusters were selected for qRT-PCR assays based on their relevance to salinity stress, and the expression analyses were carried out for different comparisons (Supplementary Tables S10 & S11). Among the 25 selected clusters, 22 clusters were evaluated for unique comparisons, whereas three clusters (49084.49984, 49084.151824, and 49084.176013) were evaluated for two different comparisons. Relative normalized expression values were compared for four clusters between C03R and T03R (Fig. 7a); five clusters between C09R and T09R (Fig. 7b); six clusters between C09L and T09L (Fig. 7c,d); three clusters between T03R and T09R (Fig. 7e); four clusters between C03L vs. C09L (Fig. 7f); and six clusters between C03R vs. C09R (Fig. 7g). The qRT-PCR results for all 25 clusters (used to study 10 downregulated and 18 upregulated cases) showed a general trend with their relative expression status as observed in RNA-seq experiments, suggesting that RNA-seq data were reliable. For multiple alfalfa clusters, similar fold-changes in gene expression were observed in RNA-seq and qRT-PCR results. For example, cluster 49084.153467, which shows high homology to high-affinity potassium transporter, showed 2.7-fold and 3.4-fold downregulations in C09L compared to T09L in RNA-seq and qRT-PCR results, respectively (Fig. 7c). But, for some alfalfa clusters, fold-changes of expression observed by qRT-PCR analyses did not show similar fold-change values observed in RNA-seq analysis. For example, cluster 49084.206430, which has high homology to boron transporter-like protein, showed 9.8-fold and 3.4-fold upregulation in C09R compared to T09R in RNA-seq and qRT-PCR results, respectively (Fig. 7b). Despite the differences in expression levels in RNA-seq vs. qRT-PCR experiments, the overall trend (upregulation or downregulation) was similar in the two experimental approaches.

## Discussion

The increasing salinity of soils and water resources, driven by natural or anthropogenic causes, has led plants to develop a series of responses and adaptive mechanisms to cope with salt-stress. In this study, we have compared the transcriptome profiles of a salt-tolerant (SISA 14-1 (G03)) and a salt-sensitive (SISA 10 (G09)) alfalfa genotype at maturity. To get an insight into the mechanisms of salt tolerance in alfalfa, high-throughput Illumina sequencing was performed on leaf and root tissues under control and salinity. Alfalfa de novo assembly was generated by combining the sequence reads derived from all the samples, and 418,480 non-redundant unigenes were obtained. The de novo assembly indicated good coverage and depth of the sequencing data. The functional annotation of unigenes against seven public databases revealed that about 83.16% of the unigenes were found in at least one database suggesting that most unigenes code for proteins (Supplementary Fig. S3). The unigenes that were not detected in any database may be lacking a known functional domain or may represent non-coding RNA genes. The unigenes were assigned to known molecular functions, biological processes, and cellular components by GO enrichment and grouped based on KEGG and KOG pathways.

Gene expression profiles were compared between the alfalfa genotypes G03 and G09, under the presence and absence of salt (Table 1), and DEGs were annotated for possible functions (Supplementary Table S4). Salt tolerance index (STI) (performance under salinity divided by performance under controlled conditions) of G03 and G09 were calculated for various morphological and physiological parameters in our previous study^5^. STI for two physiological parameters, transpiration rate (*Tr*) and stomatal conductance (*gs*), were higher in G09 (salt-sensitive) compared to G03 (salt-tolerant)^5^ (Supplementary Fig. S7). These observations suggest that enhanced salt tolerance of G03 may be a result of its increased ability to exclude excess salt from the soil. One group of proteins known to affect *gs* and *Tr* directly is aquaporins^18^. A previous study has shown that aquaporins promote salt tolerance in different species^19^. We observed that one gene that codes for aquaporin (49084.140697) was upregulated in T03L compared to C03L, and two genes that code for aquaporin proteins (49084.266975 and 49084.249391) were upregulated in T03R compared to C03R (Supplementary Table S4). On the other hand, another aquaporin gene (49084.112574) was downregulated in T09R compared to C09R (Supplementary Table S4). Upregulation of aquaporin homologs in G03 under salinity and downregulation in G09 suggests that aquaporins may be playing a positive regulatory role for salt tolerance in genotype G03. The role of aquaporins in salt tolerance may be particularly important for alfalfa, as it is moderately tolerant to salinity and can withstand salinity in irrigation water (EC_iw_) up to 16.6 dS m^−1^ under high leaching with little reduction in biomass^5^. At this high EC_iw_, in addition to ionic stress, osmotic stress is also an important component of salinity stress. Aquaporins are directly involved in osmotic regulation^20^, their induction under salinity supports their role during salinity stress in alfalfa. Some crops like almonds, grapes, strawberries, and avocados are sensitive to salinity and show a significant reduction in performance even at low salinity (EC_iw_ = 4 dS m^−1^)^3,21–24^. For these crops, this low salinity level is not high enough to trigger osmotic stress; hence aquaporins may not be directly involved in salinity tolerance in these crops.

G03 and G09 showed drastic differences in STI for shoot biomass and shoot number^5^. For STI for biomass, G03 and G09 displayed values 1.07 and 0.40, respectively. Similarly, for shoot number, the STI values were 1.28 and 0.55 for G03 and G09, respectively (Supplementary Fig. S7). These genotypes did not show significant differences in STI for plant height (Supplementary Fig. S7), suggesting that difference in biomass between two lines under salinity was primarily due to the difference in shoot number.

Tillering or lateral shoot formation is known to be regulated by several genes, including *LATERAL SUPPRESSOR* in tomato or its ortholog *MONOCULM1* (*MOC1*) in rice that encodes a GRAS family transcription factor^25,26^. The rice *moc1* mutant does not produce axillary buds leading to a single primary stem with no-tillers^25^. In our investigation, clusters 49084.169781 and 49084.74657, both encoding for GRAS family transcription factors, were induced under salinity treatment in roots of G03 and G09, respectively (Supplementary Table S4). Besides, cluster-49084.239925 was upregulated in T03L as compared to T09L. Induction of genes under salinity or expression differences between G03 and G09 may indicate the involvement of these genes in salinity tolerance. The *tillering and dwarf 1* (*tad1*) mutant in rice displays a high-tillering phenotype, as the *TAD1* gene encodes an E3 ubiquitin ligase protein that targets MOC1 for degradation in a cell-cycle dependent manner^27^. Four alfalfa *E3 ubiquitin ligase* genes (49084.176034, 49084.83258, 49084.186594, and 49084.224285) were highly upregulated in T09L compared to T03L (Supplementary Table S4). Of these, cluster-49084.224285 was also upregulated in T09R compared to T03R. These observations are perfectly in line with TAD1’s role as a negative regulator of tillering and suggest that reduced tillering in G09 under salinity may be due to higher expression of TAD1. Continuous sugar supply is critical for lateral bud growth. The rice *moc2* mutant has reduced bud growth due to disrupted fructose-1,6-bisphosphatase (FBP) activity resulting in reduced tiller number^28^. Two alfalfa genes (49084.138913 and 49048.133405) that encode for FBP were upregulated in G03 compared to G09 in leaves, both under control and salinity treatments, possibly explaining the high shoot number in G03 as compared to G09 (Supplementary Table S4). High expression of *Teosinte Branched 1* (*TB1*) is known to decrease tillering in maize^29^. TB1 encodes a TCP transcription factor and is specifically expressed in lateral buds. Analyses of TCP transcription factor homologs in alfalfa revealed that cluster 49084.152785 was downregulated in leaves of G03 under salinity compared to the control. On the other hand, 49084.181396 and 49084.232957 were upregulated in leaves and roots of G09, respectively, under salinity compared to the control (Supplementary Table S4). These observations support the negative regulation of tillering by TB1 in the G09 genotype.

Transporters and channels play vital roles in ion homeostasis for key ions like Na^+^, Cl^-^ and K^+^. Interestingly, G03 and G09 did not differ significantly in ion accumulation in shoots^5^, which suggested that the differences in the salt-tolerance abilities of these two lines may not be due to transporter proteins. Therefore, we focused our study on DEGs enriched in pathways associated with signal transduction such as hormone metabolism, calcium signaling, and redox signaling (Fig. 4–6).

Phytohormones are not only critical during plant growth and development, but the cross-talk and interplay of multiple hormone pathways also play important roles in abiotic stress tolerance, including salinity stress^30^. As expected for different types of tissues, many genes were differentially expressed in leaf vs. root comparisons (Supplementary Table S7). Our analyses of control vs. salt treatment comparisons (C03L vs. T03L, C09L vs. T09L, C03R vs. T03R, C09R vs. T09R) showed differential expressions of various genes involved in biosynthesis and signaling of auxin (IAA), ABA, cytokinin, JA, SA, and GA between salt-tolerant and salt-sensitive genotypes (Fig. 4 and Supplementary Table S7). Of the DEGs in control vs. salt treatment comparisons, the highest number was involved in JA signaling (Fig. 4), suggesting that the metabolites from the JA pathway may be crucial during salinity stress in alfalfa. For the salt-tolerant vs. salt-sensitive comparisons (C03L vs. C09L, T03L vs. T09L, C03R vs. C09R, T03R vs. T09R), the highest number of DEGs were for IAA and ethylene signaling (Fig. 4 and Supplementary Table S7), emphasizing the importance of these hormones in differential genotypic responses. Nonetheless, in both the control vs. salt and salt-tolerant vs. salt-sensitive comparisons, DEGs identified in root were significantly less than leaf (Fig. 4). These observations implied that hormonal differences might play a more important role in tissue tolerance in leaves than in roots.

Calcium signaling mechanisms are the key to regulate plant responses to both drought and salinity stresses^31^. Cytosolic Ca^2+^ serves as an important second messenger during salt stress in plants. In our analyses of the 12 comparisons, 9 displayed DEGs involved in Ca^2+^ signaling (Supplementary Table S8). As expected, based on enormous differences in the structure and function of roots and shoots, a large number of DEGs were involved in Ca^2+^ signaling in four leaf vs. root comparisons (Supplementary Table S8). For the four control vs. salt comparisons, only three DEGs were involved in Ca^2+^ signaling, suggesting that limited changes in Ca^2+^ signaling are triggered by salinity stress, whereas for the comparisons between the salt-tolerant vs. the salt-sensitive genotype, 28 DEGs were involved in Ca^2+^ signaling (Fig. 5 and Supplementary Table S8). For example, our analysis indicated a higher expression level of autoinhibited Ca^2+^-ATPase, isoform 4 (*ACA4*) like gene in C03R in comparison to C09R. Arabidopsis ACA4 has been shown to improve salt tolerance in yeast^32^. Five genes involved in Ca^2+^ signaling that encode a calcium-binding EF-hand family protein, an IQ-domain 24 containing calmodulin-binding region, an IQ-domain 33, a calreticulin, and a calcium pump ACA9, were significantly upregulated in T03L as compared to T09L (Fig. 5 and Supplementary Table S8). It would be interesting to find out the exact biological roles of these alfalfa genes during salinity stress. Nonetheless, calcium signaling may be a key contributor to provide better salt tolerance in genotype G03 than G09. It has been well established that cytosolic Ca^2+^ concentration increases in response to salt stress that subsequently activates the SOS pathway in plants, which in turn regulates Na^+^ extrusion and long-distance Na^+^ transport^33,34^. During salt stress, cytosolic Ca^2+^ also induces ROS signaling in plants in response to salt stress^35^.

ROS function as secondary messengers in response to stresses. Over accumulation of ROS (superoxide anions, hydrogen peroxide, hydroxyl radicals) cause damage to cellular structures and functions, eventually leading to cell death^36^. ROS-scavenging pathways play a vital role in maintaining a non-toxic level of ROS. Plants possess both enzymatic- and non-enzymatic antioxidative systems that play important roles in abiotic stress tolerance. Plant ROS-scavenging enzymes include superoxide dismutase, catalase, peroxiredoxin, ascorbate peroxidase (APX), glutathione peroxidase (GPX), dehydroascorbate reductase, monodehydroascorbate reductase, glutathione reductase, and glutathione S-transferase^37^. Thioredoxins are key disulfide reductase which regulate the redox status of target proteins^38^. Glutathione and ascorbic acid (vitamin C) are some of the compounds known as non-enzymatic antioxidants. Our ROS signaling pathway analysis showed that both enzymatic and non-enzymatic antioxidative/ROS-scavenging pathway genes were upregulated in G03 compared to G09 under both control (C03L vs. C09L) and salinity (T03L vs. T09L) conditions (Fig. 6 and Supplementary Table S9). There was a tenfold higher expression of a thioredoxin H-type protein (cluster-49084.166200) in T03L in comparison to T09L. In rice, overexpression of H-type thioredoxin reduced the production of H_2_O_2_ under salt stress^39^. This fact suggests that a higher expression level of thioredoxin H-type protein (cluster-49084.166200) may contribute to less production of H_2_O_2_ under salt stress in G03 than in G09. In plant cells, APX and GPX are major scavengers of H_2_O_2_. Arabidopsis APX3 and two wheat GPXs have been implicated in salinity tolerance^40,41^. We also observed that an AtAPX3-like protein (cluster-49084.145718) and a GPX protein (cluster-49084.134058) were upregulated in T03L compared to T09L suggesting their protective role against salt-induced oxidative stress in G03 than G09 (Fig. 6 and Supplementary Table S9). Glutathione reductase (GR) is also an important antioxidant enzyme in plants and in shown to regulate salt tolerance in rice^42^. We observed a higher expression of a GR (cluster-49084.131473) in T03L than in T09L (Fig. 6 and Supplementary Table S9). It should be noted that we did not find the upregulation of any redox signaling genes in T09L in comparison to T03L. Our findings indicate that G03 plants are better equipped to tolerate salt-induced oxidative stress. Altogether, current findings suggest that the redox signaling pathway positively regulates salt tolerance in alfalfa genotype G03 compared to G09.

Validation of RNA-seq results using qRT-PCR showed a high level of consistency between the two experiments, supporting the authenticity of the RNA-seq data (Fig. 7). In some cases, there were inconsistencies in the magnitude of the changes observed for gene expression between the two approaches. One reason for these inconsistencies could be the differential sensitivity of RNA-seq and qRT-PCR. In addition, it is worth noting that the magnitude of changes observed in RNA-seq and qRT-PCR results varied primarily when read counts were less than ~ 200 in RNA-seq results. These findings suggest that genes with high read counts should be considered for future validation studies.

## Methods

### Plant material and salt treatment

In a previous study, we subjected 12 alfalfa genotypes to irrigation waters of electrical conductivity (EC_iw_) of 1.97 dS m^−1^ (control salinity) and 16.6 dS m^−1^ (high salinity) and characterized the genotypes based on their biomass accumulation, shoot ion concentrations, physiological parameters and gene expression^5^. The most salt-tolerant genotype, SISA 14-1 (G03), and the most salt-sensitive genotype, SISA 10 (G09), were selected for transcriptome analysis. The experiment was conducted at the United States Salinity Laboratory (USDA-ARS), Riverside, CA, in a greenhouse lysimeter system with day/night temperatures of 28 °C/18 °C, under natural illumination. Plants were grown in randomized sand tanks (120 cm long × 60 cm wide × 50 cm deep) in three replications. Irrigation solutions were prepared in 890 L reservoirs under the greenhouse and pumped through PVC pipes for irrigation. Excess water drained back to the reservoirs by gravity. The water lost due to evapotranspiration was replenished, and EC of irrigation water was maintained. Salinity treatment was initiated 7 days after clones were transplanted. Plants were irrigated once a day with a basic nutrient solution containing the following mineral ions and concentrations: 1.95 mmol_c_ L^−1^ Cl^-^, 1.88 mmol_c_ L^−1^ Na^+^, 5.0 mmol_c_ L^−1^ NO_3_^-^, 0.5 mmol_c_ L^−1^ PO_4_^2-^, 5.59 mmol_c_ L^−1^ K^+^, 4.4 mmol_c_ L^−1^ Ca^2+^, 4.15 mmol_c_ L^−1^ Mg^2+^, 4.25 mmol_c_ L^−1^ SO_4_^2^-, plus the following micronutrients: 50 µmol L^−1^ Fe, added as Fe-DTPA (Sprint 330), 1.2 µmol L^−1^ ZnSO_4_.7H_2_O, 0.3 µmol L^−1^ CuSO_4_.5H_2_O, 0.1 µmol L^−1^ (NH_4_)_6_Mo_7_O_24_.4H_2_O, 23 µmol L^−1^ H_3_BO_3_, and 15 µmol L^−1^ MnSO_4_. The high-salinity water treatment contained 128.95 mmol_c_ L^−1^ Cl^-^, 149.1 mmol_c_ L^−1^ Na^+^, 65 mmol_c_ L^−1^ SO_4_^2−^, 3.0 mmol_c_ L^−1^ NO_3_^−^, 0.5 mmol_c_ L^−1^ PO_4_^2−^, 3.5 mmol_c_ L^−1^ K^+^, 24.9 mmol_c_ L^−1^ Ca^2+^, 22.35 mmol_c_ L^−1^ Mg^2+^. To avoid osmotic shock caused by the abrupt increase in salinity, irrigation water salinity was increased stepwise over 14 days to reach 16.6 dS m^−1^. The experiment was carried out for 20 months.

### RNA extraction and RNA sequencing

Leaf and root tissues were taken from plants irrigated for 20 months with either control or high-salinity water. Three biological replicates of both cultivars and treatments were used for RNA sequencing. Total RNA was extracted from root and leaf samples using TRIzol reagent (Invitrogen, Carlsbad, CA, USA). In order to remove any DNA contamination, RNA was treated with DNase I following the manufacturer’s instructions (Thermo Scientific, Waltham, MA, USA). RNA quantity and quality were determined with an Agilent 2100 Bioanalyzer (Agilent Technologies, USA) and Nanophotometer (IMPLEN, CA, USA). Sequencing libraries were generated using the NEBNext Ultra RNA library prep kit for Illumina (NEB, Ipswich, MA, USA). Poly-T oligo-attached magnetic beads were used to purify mRNA from total RNA, the cleaved RNA fragments were transcribed into the first-strand cDNA using random hexamer primers and MM-MuLV Reverse Transcriptase (RNase H^-^) with second-strand cDNA synthesis being subsequently performed using DNA polymerase I and RNase H. The fragments were ligated to NEBNext adaptors with a hairpin loop structure. The clustering of the index-coded samples was performed on a cBot Cluster Generation System using HiSeq PE Cluster Kit cBot-HS (Illumina). After clustering, the libraries were sequenced on an Illumina HiSeq platform and paired-end reads of 150 bp were generated (Novogene Corp. Inc., Sacramento, CA).

### De novo transcriptome assembly and functional annotation

In-house perl scripts were used to clean the raw reads by removing reads containing adapter, poly-N, and low-quality reads. Quality score Q < 20, Q < 30, GC-content, and sequence duplications of the clean data were also calculated. All the downstream analyses were based on clean, high-quality, data. All left reads were pooled together in a single left.fq file and similarly all right reads were pooled in a single right.fq file. The de novo transcriptome assembly was performed by Trinity^43^ with a min_kmer_cov set to 2 and all other parameters set to default. Trinity is a novel method for efficient and robust de novo reconstruction of transcriptomes from RNA-Seq data; it partitions the sequence data into many de Bruijn graphs, each representing the transcriptional complexity at a given gene, and then independently processes each graph to extract the full-length splicing isoforms. To assemble the RNA-Seq data more efficiently, we first normalized the reads according to the depth of sequencing coverage using the tools included in the Trinity^49^ software distribution. To achieve comprehensive functional annotation, BLAST 2.2.28 + ^44^, HMMER 3.0, Blast2GO^45^, Kyoto Encyclopedia of Genes and Genomes (KEGG)^46^ and KEGG Automatic Annotation Server (KAAS)^47^ were used to provide information from seven databases: NR (NCBI non-redundant protein sequences), NT (NCBI non-redundant nucleotide sequences), Pfam (Protein family)^48^, KOG/COG (Clusters of Orthologous Groups of proteins), Swiss-Prot (a manually annotated and reviewed protein sequence database), KO (Kyoto Encyclopedia of Genes and Genomes Ortholog database)^49^ and GO (Gene Ontology database).

### Differential gene expression

The cleaned raw reads data were mapped onto the assembled transcriptome and a read count for each transcript was obtained from the mapping results. The gene expression levels were estimated using RSEM^50^. RSEM (RNA-Seq by Expectation–Maximization) is a statistical tool for performing gene and isoform level quantification, and its algorithm is based on the expectation–maximization technique which is represented by the directed graphical model. To perform differential expression analysis of two conditions/groups, we used a DESeq2 R package (1.10.1), which provides statistical routines based on the negative binomial distribution model for identifying differential expression in digital gene expression data. The resulting *P* values were adjusted using the Benjamini and Hochberg’s approach to control false discovery rate^51^. Genes with an adjusted *P*-value ≤ 0.05 (*q*-value) found by DESeq2 were considered as differentially expressed. Both the *q*-value ≤ 0.05 & |log_2_ (fold-change)|≥ 2 was set as the threshold for significant differential expression.

### GO enrichment analysis of differentially-expressed genes (DEGs)

GO analysis of the DEGs was implemented by the GOseq R package based on Wallenius’ non-central hypergeometric distribution, which can adjust for gene length bias in the DEGs. Mathematically, it is indisputable that all commonly used criteria for judging differential expression interact with the gene length and read count; this justifies why the gene length bias exists and so needs to be accounted for. The GOseq method accounts for all such biases. The GO distribution was performed for all three GO terminologies: biological process, cellular component, and molecular function. One of the significant uses of gene ontology is to carry out enrichment studies on gene sets, i.e. a set of genes that are up- or down-regulated under certain circumstances; an enrichment assessment will find out which GO terms are over-represented or under-represented using gene-set annotations. GO terms annotated from all the DEGs were used as a study set and GO terms annotation from all the unigenes and set as references in Blast2GO to perform the Fisher’s Exact test using an FDR cutoff of 0.05^45^. All the adjusted statistically significant values of the GO terms were negative 10‑base log transformed.

### KEGG pathway enrichment analysis of DEGs

KEGG is a database resource for understanding high-level functions and utilities of the biological system, such as the cell, the organism and the ecosystem, from molecular-level information, especially large-scale molecular datasets generated by genome sequencing and other high-throughput experimental technologies^46^ (http://www.genome.jp/kegg/). We used KOBAS software to test the statistical enrichment of unigenes in KEGG pathways^49^. For the KEGG enrichment of the DEGs, we used clusterProfiler tool^52^. clusterProfiler is a R package that automates the process of enrichment analysis for gene clusters and the classification of biological terms. It uses hypergeometric distribution to perform the enrichment tests, and to prevent high false discovery date (FDR) in multiple testing, *q*-values are also estimated for FDR control.

### Functional analysis and visualization

MapMan (https://mapman.gabipd.org/) visualizations were performed for the functional analysis and to efficiently display our genomic datasets for comparative analysis^53^. Contigs were classified into a set of functional hierarchical classifications (BINs) using Mercator with a BLAST cutoff of 50^53^. Because one unigene could have various contigs, the functional term ‘unigene’ was derived from its representative contig that had the highest bit score.

### Quantitative reverse transcription-PCR (qRT-PCR)

Twenty-five differentially expressed clusters of alfalfa were selected based on their predicted biological function related to salinity stress (Supplementary Table S11). BLAST analyses were performed for each selected cluster using the Phytozome portal (v12.1) to identify the *M. truncatula* gene that has the highest sequence homology. Subsequently, the genomic sequence of the *M. truncatula* gene was compared against the corresponding cluster sequence to identify the intron–exon boundaries of the alfalfa gene. At least one primer out of each pair was designed from two exons flanking an intron (Supplementary Table S10).

We performed qRT-PCR assays as described previously^5^. The RNA samples that were used for RNA-seq experiments were also used for qRT-PCR analyses. DNase I treatment was performed on all RNA samples to remove contaminating DNA, according to the manufacturer’s instructions (Thermo Scientific, Waltham, MA, USA). A BioRad CFX96 System was employed to perform qRT-PCR experiments using iTaqTM Universal SYBR® Green One-Step Kit (Bio-Rad Laboratories, Hercules, CA, USA). The reactions were carried out in a total volume of 10 μl containing 20 ng total RNA, 0.125 μl iScript™ Reverse Transcriptase, 5 μl of 2 × one-step SYBR® Green Reaction mix, and 0.75 μM of each of the primers. The following PCR conditions were used: 50 °C for 10 min, 95 °C for 1 min, then 40 cycles of 95 °C denaturation for 10 s, 57 °C annealing for 30 s, and 68 °C extension for 30 s. All qRT-PCR assays were performed using RNA samples from three biological replicates and two technical replicates. To normalize expression among plates three samples were used as inter-plate controls. Two alfalfa genes, *Actin* (*Act*) and *glyceraldehyde 3-phosphate dehydrogenase* (*G3PD*) were used as reference in our expression analysis. To identify differentially expressed genes, the cycle threshold values of each gene were compared to the reference, and differences in relative expressions were calculated.

## Supplementary Information

Supplementary Table S1.

Supplementary Table S2.

Supplementary Table S3.

Supplementary Table S4.

Supplementary Table S5.

Supplementary Table S6.

Supplementary Table S7.

Supplementary Table S8.

Supplementary Table S9.

Supplementary Table S10.

Supplementary Table S11.

Supplementary Figures.

## Data Availability

All the sequencing reads generated from Illumina HiSeq RNA-Seq are available in NCBI SRA: SRR9949086 to SRR9949093 (https://www.ncbi.nlm.nih.gov/Traces/study/?acc=SRP218060&o=acc_s%3Aa). All other datasets supporting this study are included in the article and its supplementary material.
