# Supplementary material for: Transcriptional profiling of two contrasting genotypes uncovers molecular mechanisms underlying salt tolerance in alfalfa
Source: Sci Rep. 2021 Mar 4;11:5210. doi: 10.1038/s41598-021-84461-w (PMC7933430; doi:10.1038/s41598-021-84461-w)
Supplement: Supplementary file 12 — Supplementary Figures. [file 41598_2021_84461_MOESM12_ESM.pdf]

Transcriptional profiling of two contrasting genotypes uncovers molecular mechanisms underlying salt tolerance in alfalfa

Rakesh Kaundal, Naveen Duhan, Biswa R. Acharya, Manju V. Pudussery, Jorge F.S. Ferreira, Donald L. Suarez, Devinder Sandhu

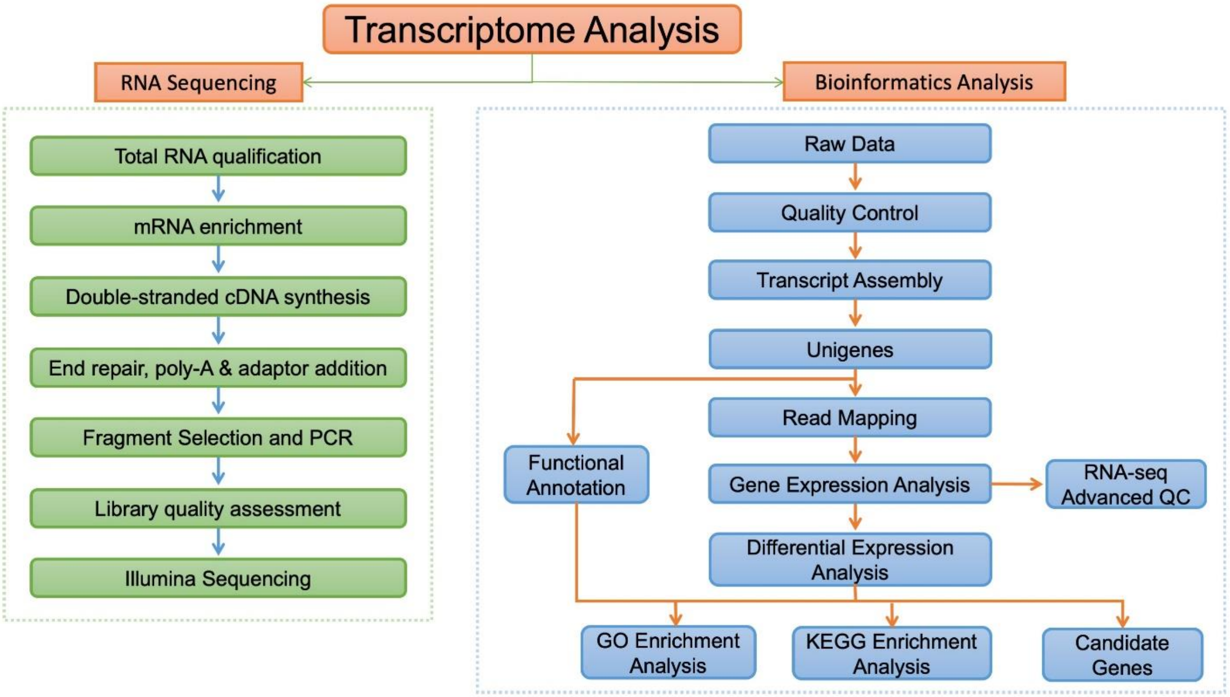

Supplementary Fig. S1. The overall workflow of the transcriptome analysis of two alfalfa genotypes in response to salinity stress.

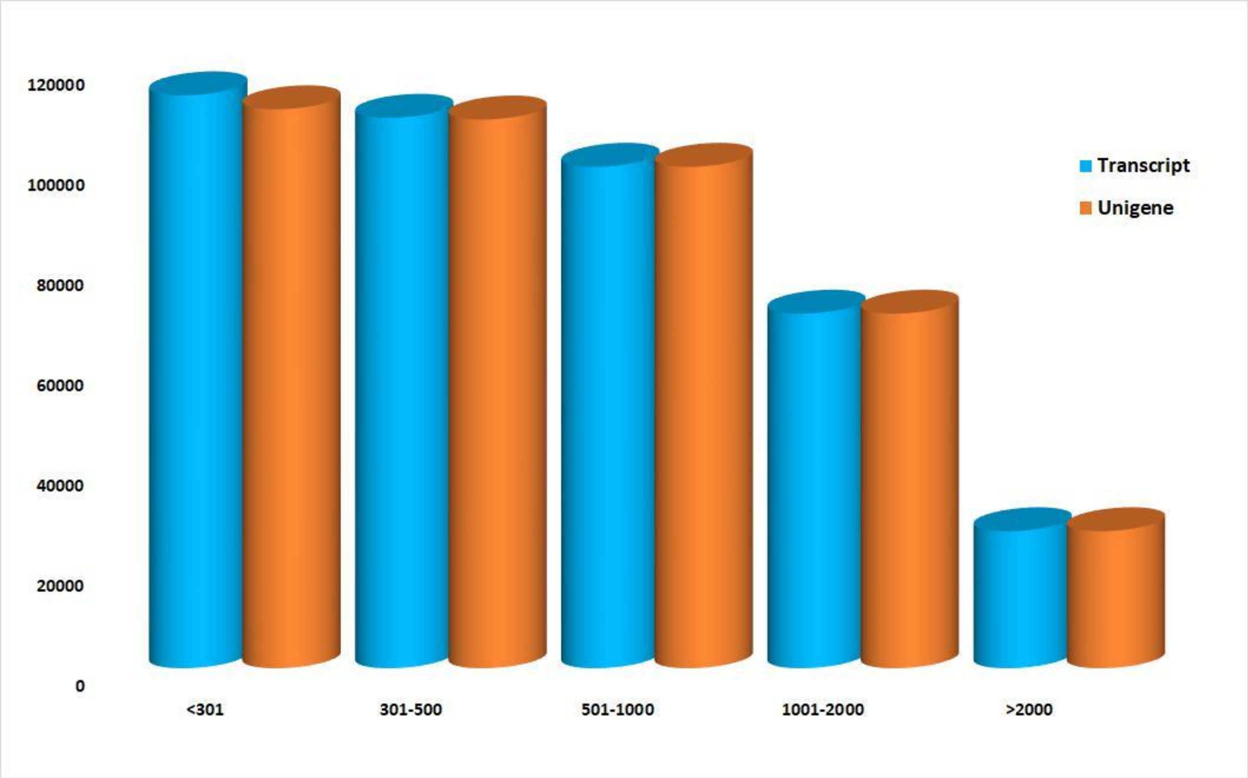

Supplementary Fig. S2. Representation of transcripts and unigenes in alfalfa. Size of transcripts and unigenes are shown in *x-axis* and numbers are in *y-axis*.

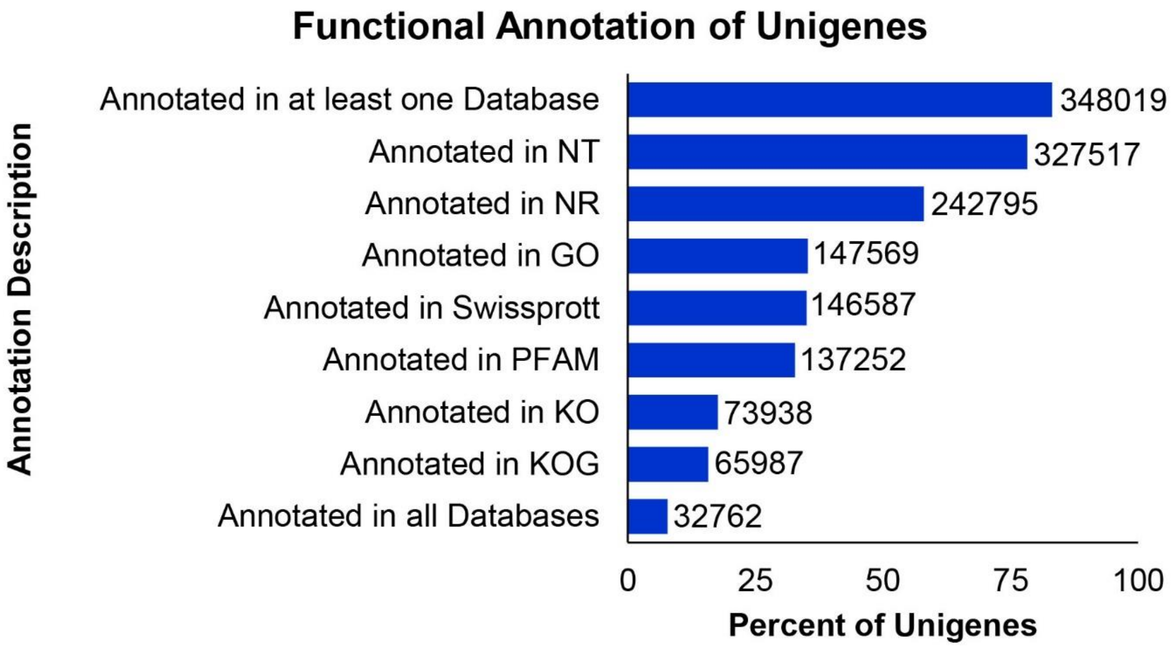

Supplementary Fig. S3. Functional annotation statistics of the alfalfa transcriptome assembly. NT, NCBI non-redundant nucleotide sequences; NR, NCBI non-redundant protein sequences; GO, gene ontology, SwissProt, a manually annotated and reviewed protein sequence database; PFAM (Protein family); KO (Kyoto Encyclopedia of Genes and Genomes Ortholog database); KOG (EuKaryotic Orthologous Groups).

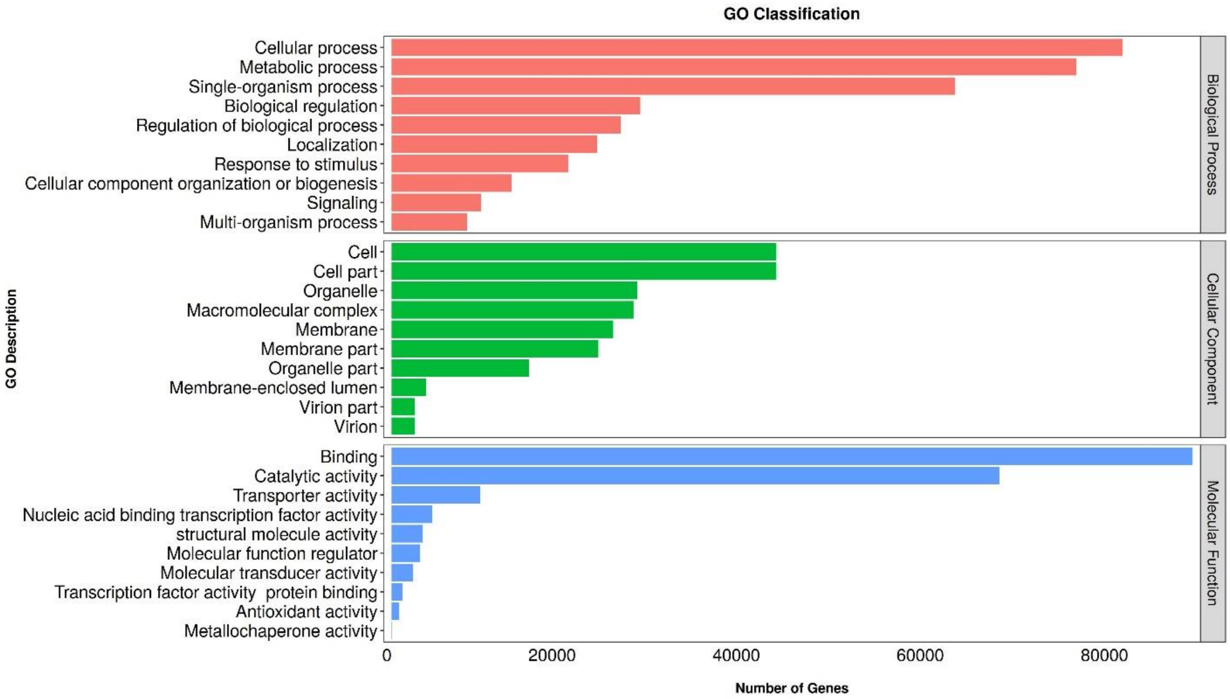

Supplementary Fig. S4. Gene Ontology (GO) distribution of the alfalfa transcriptome assembly. Top 10 GO terms for each subclass (Biological Process, Cellular Component, and Molecular Function) are shown.

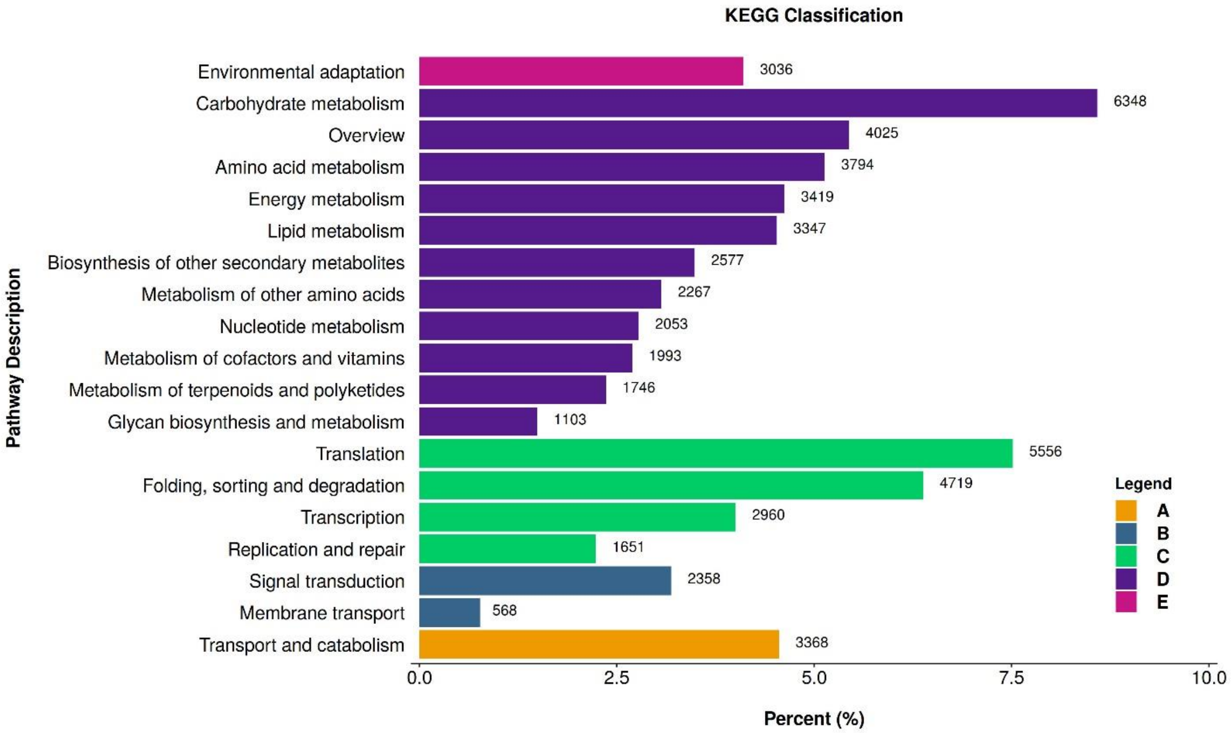

Supplementary Fig. S5. Representation of KEGG pathways distribution for transcriptome assembly. The unigenes are further classified into metabolic categories and represented as percentage subcategory of the unigenes. The total number of unigenes are represented on the right of each subcategory. A) Cellular process pathway. B) Environmental information processing pathways. C) Genetic information processing pathways. D) Metabolic pathways. E) Organism system pathway.

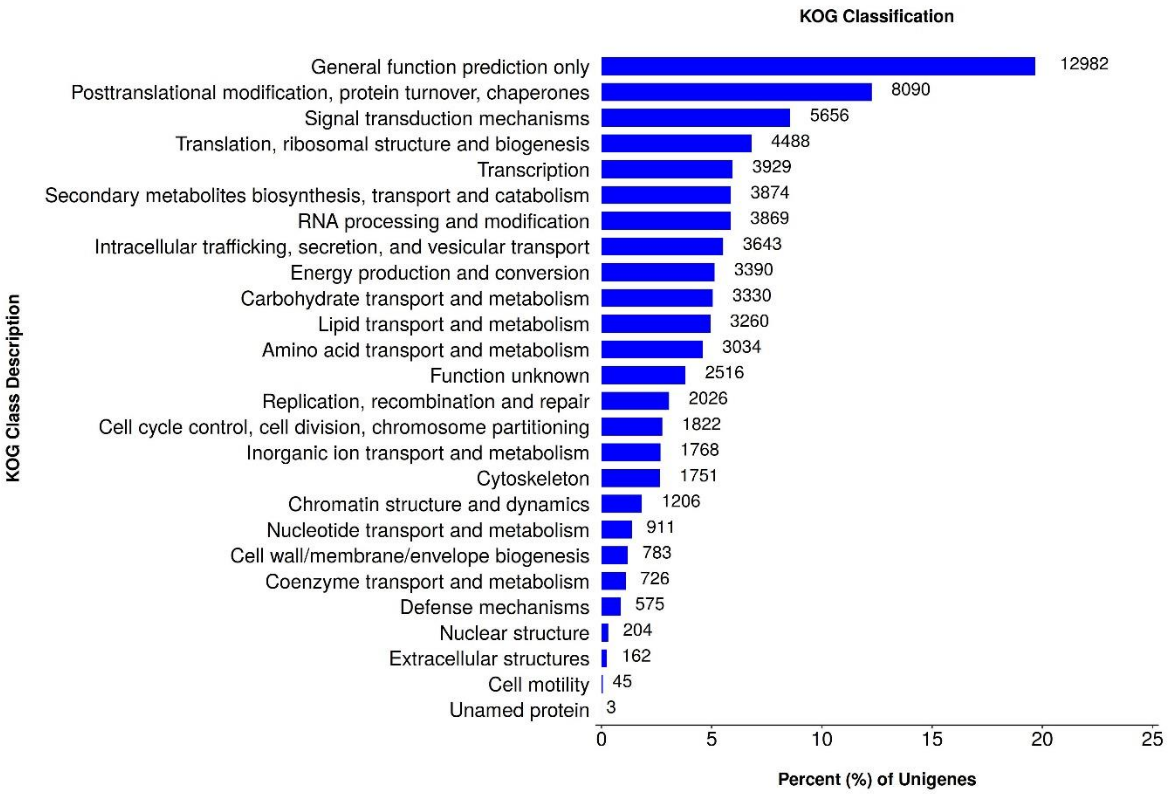

Supplementary Fig. S6. EuKaryotic Orthologous Groups (KOG) classification representation of transcriptome assembly.

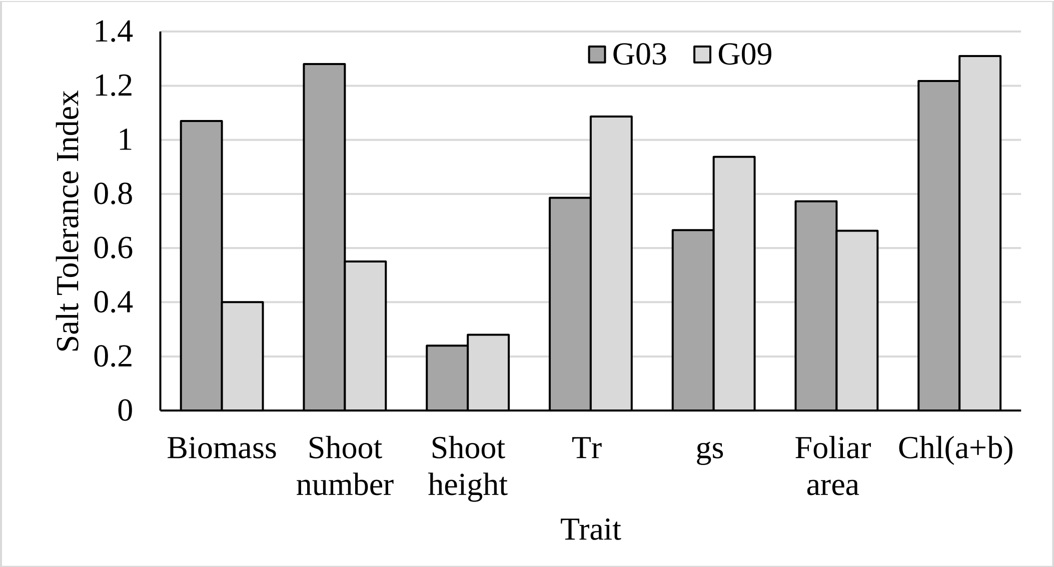

Supplementary Fig. S7. Comparison of salt tolerance index (STI) for various traits between G03 (salt-tolerant) and G09 (salt-sensitive) genotypes[5].
